# Calycosin Orchestrates Osteogenesis of Danggui Buxue Tang in Cultured Osteoblasts: Evaluating the Mechanism of Action by Omics and Chemical Knock-out Methodologies

**DOI:** 10.3389/fphar.2018.00036

**Published:** 2018-02-01

**Authors:** Amy G. W. Gong, Ran Duan, Huai Y. Wang, Tina T. X. Dong, Karl W. K. Tsim

**Affiliations:** ^1^HKUST Shenzhen Research Institute, Shenzhen, China; ^2^Division of Life Science and Center for Chinese Medicine, The Hong Kong University of Science and Technology, Hong Kong, Hong Kong

**Keywords:** Astragali Radix, Angelicae Sinensis Radix, Danggui Buxue Tang, RNA-seq, metabolomics, osteoblast

## Abstract

Danggui Buxue Tang (DBT), an ancient Chinese herbal decoction commonly used to mitigate menopausal osteoporosis, contains two herbs: Astragali Radix (AR) and Angelicae Sinensis Radix (ASR). The exact efficacy of individual chemical(s) within DBT, or in any herbal mixture, is hard to be revealed. Calycosin and ferulic acid have been reported to be the predominant chemicals found within DBT, and its roles in regulating osteoblastic differentiation have been proposed here. To probe the roles of calycosin and ferulic acid, these chemicals were specifically depleted from the DBT extracts. Here, calycosin-depleted DBT (DBT_Δcal_) and ferulic acid-depleted DBT (DBT_Δfa_), generated by semi-preparative HPLC, were coupled with RNA-seq and metabolomics analyses to reveal the synergistic functions of individual chemicals within a complex herbal mixture. The expressions of osteogenic differentiation markers were significantly increased under the treatments of DBT and DBT_Δfa_. The DBT-induced genes were markedly reduced in the absent of calycosin, i.e., DBT_Δcal_. In cultured osteoblasts, the DBT-activated Wnt/β-catenin and MAPK/Erk and signaling pathways were greatly affected when calycosin was depleted. By metabolomics analysis in DBT-treated osteoblasts, the profile of metabolites triggered by DBT_Δcal_ showed distinction to that of DBT and/or DBT_Δfa_. Thus, our findings indicated that calycosin, rather than ferulic acid, could be an indispensable chemical in DBT to orchestrate multi-components of DBT in achieving maximal osteogenic properties.

## Introduction

Osteoporosis is one kind of bone diseases with reducing bone mineral density and elevated risk of fracture (Raisz, 2005). The reduced activity of osteoblastic differentiation is one of early symptoms of osteoporosis (Ota et al., 2016). This disease is one of the most common syndromes found within menopausal women. Estrogen replacement therapy (ERT) is widely utilized for mitigating osteoporosis as well as to ease other menopausal symptoms (Na et al., 2014; Khan et al., 2017). Nevertheless, the side effects of ERT have been reported, i.e., possibly causing breast cancer and cardiovascular disease (Eden, 2013; Chuffa et al., 2017). Phytomedicine is a popular choice for women to reduce the menopausal symptoms. Amongst botanical drugs, traditional Chinese medicine (TCM) draws attention from the world because of its historical records of clinical efficacies and safety (Tan et al., 2016).

Synergistic properties and compatibility of different ingredients are the basic principles of TCM. However, the correlations between bioactive chemicals within a herbal decoction and the detailed underlying mechanisms have not been elucidated, and which hinder the acceptance of TCM by general public. Danggui Buxue Tang (DBT) is one of the simplest herbal formulae, containing two herbs: Angelicae Sinensis Radix (ASR) and Astragali Radix (AR) at the ratio of 1:5 (Dong et al., 2006; Choi et al., 2011; Zhang et al., 2012). DBT was recorded in *Neiwaishang Bianhuo Lun* by Li Dongyuan in Jin dynasty (about AD 1247). Nowadays, DBT is suggested to be taken every day as a remedy for symptoms of menopause, i.e., osteoporosis (Gao et al., 2007; Lin et al., 2017).

To reveal the mechanistic action of TCM therapy, transcriptomics and metabolomics are good tools, and they have been employed to identify and quantify gene expressions and related metabolites (Wang et al., 2009). Indeed, the studies on gene expressions, triggered by bioactive substances, within DBT are still insufficient. Calycosin, a major chemical in AR, as well as in DBT, showed abilities to suppress the RANKL-mediated osteoclastogenesis in cultured bone marrow macrophages (Quan et al., 2015). Ferulic acid, the most abundant bioactive chemical found in ASR, is one of promising natural chemicals within DBT to suppress reactive oxygen species (ROS) formation (Gong et al., 2015, 2016). Both calycosin and ferulic acid were selected to be the targets to perform specific chemical knock-out from DBT. Calycosin-depleted DBT (DBT_Δcal_) and ferulic acid-depleted DBT (DBT_Δfa_) were generated by semi-preparative HPLC. Therefore, we would like to employ methods of transcriptomics and metabolomics to analyze signaling pathways and metabolites triggered by DBT, as well as its chemical knock-out DBT. The identified distinct genes or metabolites, regulated by these herbal decoctions, could serve as active biomarkers in quality control of the herbal decoction, and subsequently those identified genes/metabolites could provide comprehensive and detailed analysis for solving the mechanism of multi-components of TCM.

## Materials and Methods

### Herbal Decoction Preparation

The roots of 3-year-old *Astragalus membranaceus* (Fisch.) Bunge var. *mongholicus* (Bunge) Hsiao (AR) from Shanxi Province (Ma et al., 2002) and 2-year-old *Angelica sinensis* (Oliv.) Diel roots (ASR) from Minxian of Gansu Province (Zhao et al., 2003) were collected in 2013. The authentication of plant materials was identified morphologically by Dr. Tina Dong at Hong Kong University of Science and Technology (HKUST). The voucher specimens (voucher # 02-9-1 for ASR and voucher # 02-10-4 for AR) were deposited in the Centre for Chinese Medicine of HKUST. In preparing DBT, exact amounts of AR and ASR were weighed according to a ratio of 5:1 and then mixed well. The mixture was boiled in eight volumes of water (v/w) for 2 h, and the extraction was repeated twice (Song et al., 2004; Dong et al., 2006; Choi et al., 2011). The extracts were dried by lyophilization and stored at -80°C. To prepare DBT_Δcal_ and DBT_Δfa_, a Dikma, Diamonsil C18 column (10.0 mm × 250 mm, 5 μm) was used to deplete calycosin or ferulic acid from DBT. The depletion methods and conditions were described previously (Gong et al., 2015, 2016). The authentications of DBT_Δcal_ and DBT_Δfa_ were revealed by LC–MS/MS (Gong et al., 2015, 2016). All the samples, collected by chemical-depletion method, were lyophilized and re-dissolved in water for biological test and analytic measurement.

### Anthrone-Sulfuric Acid Detection Method

The herbal extracts were precipitated in 6 volumes of 75% ethanol incubated for 24 h at 4°C, and then centrifuged at 4000 × *g* for 30 min. The precipitate was re-dissolved in water, dried by lyophilization and stored at -80°C. The anthrone-sulfuric acid method was used to quantify polysaccharide. Two grams of dextran was dissolved in 10 mL of water to give serial concentrations (at 2, 10, 20, 40, and 100 times of dilutions). Standard solution (0.6 mL), or prepared sample, was adjusted to 1.0 mL final volume. Then, 4.0 mL of freshly prepared 0.2% anthrone-sulfuric acid was added. Afterward, the solution was incubated in boiled water for 10 min and cooled down. Absorbance at 630 nm was measured.

### Osteoblast Culture

The experimental procedures had been approved by The Animal Experimentation Ethics Committee of the Hong Kong University of Science and Technology (No. 17-283 for Animal Ethics Approval) and under the guidelines of “Principles of Laboratory Animal Care” (NIH publication No. DH/HA&P/8/2/3). Rat primary osteoblasts were prepared and cultured by the method described by Orriss et al. (2009) with minor modifications (Choi et al., 2011). In brief, postnatal day 1 rats were decapitated to collect calvarias. Tissues were sequentially digested by 1% trypsin for 10 min, 0.2% collagenase for 20 min and 0.2% collagenase for further 45 min. After the digestion, the supernatant was collected and centrifuged at 1,500 rpm for 5 min. Osteoblastic cells were re-suspended and maintained in MEMα, supplemented with 10% FBS, 2 mM L-glutamine and 1% PS. All culture medium and its reagents were purchased from Invitrogen (Waltham, MA, United States).

### Mineralization Assay

In mineralization analysis, osteoblasts were cultured for 21 days. The treatment of drugs and β-glycerolphosphate (5 mM; Sigma–Aldrich, St. Louis, MO, United States) were performed in a 3-day interval. After 21 days of culturing, the cells were rinsed with de-ionized water twice and fixed in 70% ice-cold ethanol for 1 h at 4°C. Mineralization assay was performed by using Alizarin Red, in which the cells were stained for 15 min at room temperature and washed 5X with deionized water. The stained cells were then dehydrated with 70% ethanol followed by absolute ethanol. Orange red staining indicated the position and intensity of calcium deposits.

### Alkaline Phosphatase Assay

Cultured cells in 24-well plate were treated with drugs for 7 days. Cultures were then collected by lysis buffer containing 0.2% Triton X-100 (pH 7.8), 1 mM dithiothreitol and 100 mM potassium phosphate. Alkaline phosphatase activity in cultured cells was measured by mixing the cell extract with 5 mM *p*-nitrophenyl phosphate in a buffer (pH 10.4) containing 0.1 M glycine, 1 mM MgCl_2_ and 1 mM ZnCl_2_ at 37°C. After incubation at 37°C for 3 h, the absorbance was measured at 405 nm. The enzyme activity was expressed as micromole of cleaved substrate per milligram of protein.

### RNA Sequencing Analysis

Cultures of rat osteoblasts were treated with 1 mg/mL of DBT, DBT_Δcal_, or DBT_Δfa_ for 7 days. Total RNAs were isolated by RNAzol reagent (Invitrogen). Total RNAs were subjected to RNA sequencing analysis (BGI, Ltd., Shenzhen, China) to determine the differential gene expressions in cultured cells. Gene expression was calculated using the FPKM method (fragments per kilobase transcriptome per million reads) (Trapnell et al., 2010). GO enrichment was performed using DAVID^1^ or KEGG^2^ (Huang et al., 2009).

### PCR Analysis

Primary cultures of rat osteoblasts, were treated with 1 mg/mL of DBT, DBT_Δcal_, or DBT_Δfa_ for 7 days. Total RNAs were isolated by RNAzol reagent (Invitrogen), and 5 μg of total RNA was reverse-transcribed by Moloney Murine Leukemia Virus Reverse Transcriptase (Invitrogen), according to the manufacturer’s instructions. Qualitative PCR was performed to determine the expression of Runx2, osteonectin, osteocalcin and collagen type 1 α 1 (Col1a1). The primers were: col1a1: 5′-TCC TGC CGA TGT CGC TAT C-3′ and 5′-CAA GTT CCG GTG TGA CTC GT-3′; osteonectin: 5′-TCT CTG CTC ACT CTG CTG G-3′ and 5′-GTG GTG CCA TAG ATG CGC T-3′; osteocalcin: 5′-GAA GAG ATG GTG GCG GAG-3′ and 5′-ACA GGC AGG GGG CAA TGT ATT TG-3′; Runx2: 5′-AAC TTC CTG TGC TCC GTG CT-3′ and 5′-GAC TGT TAT GGT CAA GGT GAA-3′; Glyceraldehyde 3-phosphate de-hydrogenase (GAPDH) was used as an internal control, and its primer sequences were 5′-AAC GGA TTT GGC CGT ATT GG-3′ and 5′-CTT CCC GTT CAG CTC TGG G-3′. Transcript levels were quantified by using ΔCt value method, where the values of target genes were normalized by GAPDH in the same sample at first before comparison. PCR products were analyzed by gel electrophoresis and melting curve analysis to confirm the specificity.

### Transfection of Primary Cultured Osteoblasts

Cultured osteoblasts were transfected with pRunx2-Luc DNA construct by Lipofectamine 3000 transfection reagent, as described in the manufacture’s instruction, separately. The transfection was performed 24 h after the cells were sub-cultured. Before transfection, change 450 μL fresh medium for each 24-well plate. Twenty μL of Lipofectamine 3000 reagent and 8 μL of DNA construct containing 10 μg of DNA construct were diluted by Opti-MEM, respectively. Mixed well and incubated at room temperature for 10 min before adding to the wells. The transfection efficiencies were about 20%, as determined by another control plasmid of containing β-galactosidase, under a cytomegalovirus enhancer promoter, with the method described previously (Lim and Chae, 1989).

### Luciferase Assay

Cells were seeded in 24-well plate and treated with drugs for 7 days. Cells were solubilized in lysis buffer (100 mM potassium phosphate buffer, pH 7.8, containing 1 mM DTT) immediately. The cell lysate was centrifuged in 1,500 × *g* for 5 min in 4°C, 50 μL of the supernatant was transferred to the assay plate and set on the luminance reading machine (FLUO Star OPTIMA). The readings of luminance intensity were equalized by the protein concentration of lysates, and the data indicated to the luciferase activities of the samples.

### Translocation Assay

Cultures were treated with drugs for different durations. After drug treatment, including all the inhibitors or activators, the cultures were washed by 1X PBS for twice, and then using Qproteome Nuclear Protein Kit (Qiagen, Hilden, Germany) to extract nuclear and cytosol separately, according to the manufacturer’s instruction. Twenty-five μL of 0.15 μg/μL nuclear extract of β-catenin was dissolved in lysis buffer (0.125 M Tris-HCl, pH 6.8, 4% SDS, 20% glycerol, and 2% 2-mercaptoethanol). The lysate was denatured at 95°C for 5 min twice and then subjected to SDS-PAGE analysis. After transferring the proteins to membranes, the membranes were incubated with different antibodies at various dilutions. Anti-β-catenin (∼95 kDa) at 1: 5,000 dilutions, anti-histone 1 (∼30 kDa) at 1: 5,000 dilutions were kept at 4°C for 12 h. Following incubation in horseradish peroxidase (HRP)-conjugated anti-rabbit secondary antibodies in 1: 5,000 dilutions for 3 h at room temperature, the immune-complexes were visualized by an enhanced chemiluminesence (ECL) method (Amersham Biosciences, Piscataway, NJ, United States). Antibodies were from Cell Signaling (Danvers, MA, United States). The band intensities in the control and agonist-stimulated samples, run on the same gel and under strictly standardized ECL conditions, were compared on an image analyzer, using in each case a calibration plot constructed from a parallel gel with serial dilutions of one of the samples.

### Phosphorylation Assay

The phosphorylation assay was determined by western blot assay. Cultures were serum-starved for 3 h before the herbal or activator application. After drug treatment, including all activators, the cultures were collected immediately in lysis buffer, and the protein were subjected to SDS-PAGE analysis. After transferring the proteins to membranes, the membranes were incubated with anti-GSK 3β (Ser 9) (Cell Signaling) and anti-ERα (Ser 118) (Cell Signaling) at 4°C for 12 h. Following incubation in HRP-conjugated anti-rabbit secondary antibodies in 1: 5,000 dilutions for 3 h at room temperature, the immune-complexes were visualized by the ECL method (Amersham Biosciences). The band intensities in the control and agonist-stimulated samples, run on the same gel and under strictly standardized ECL conditions, were compared on an image analyzer, using in each case a calibration plot constructed from a parallel gel with serial dilutions of one of the samples.

### Metabolites Analysis

Cultured osteoblasts were treated with 1 mg/mL of various herbal extracts for 7 days. After drug treatments, the cell lysate was harvested by methanol, sonicated for 30 min, centrifuged for 10 min, and the supernatant was performed LC–MS/MS analysis. The mobile phase was composed of 0.1% formic acid in acetonitrile (A) and 0.1% formic acid in water (B) using the following gradient program: 0–5 min, isocratic gradient 10.0–10.0% (A); 5–15 min, linear gradient 10–35% (A); 15–20 isocratic gradient 35–35%. The flow rate was 0.4 ml/min. The column temperature was at 25°C. The injection volume was 5 μL.

### Statistical Analysis and Other Assays

Protein concentrations were measured by Bradford’s method (Hercules, CA, United States). Heat map was performed by Clustering (version 3.17.1). Statistical tests have been done by using one-way analysis of variance. Data were expressed as Mean ± SEM, where *n* = 3–5. Statistically significant changes were classified as significant (^∗^) where *p* < 0.05, more significant (^∗∗^) where *p* < 0.01 and highly significant (^∗∗∗^) where *p* < 0.001. Principal component analysis (PCA) of the relative peak areas and retention time were performed using SPSS for Windows 16.0 software (SPSS Corporation, Armonk, NY, United States) to evaluate the difference of groups of samples.

## Results

### Chemical and Pharmacological Profiles of DBT, DBT_Δcal_, and DBT_Δfa_

A well-standardized herbal decoction was to ensure the consistency and repeatability of drug treatment in bioassay. The quality controls of DBT, DBT_Δcal_, and DBT_Δfa_ were performed on a HPLC (**Supplementary Figure S1A**), as reported previously (Gong et al., 2015). To ensure a complete recovery after HPLC column, the DBT extracts, before and after HPLC separation, were compared. Both DBTs showed a close chemical similarity (**Supplementary Figure S1B**). Re-added calycosin back to DBT_Δcal_ recovered fully the osteogenic functions (**Supplementary Figure S1C**). In one gram of DBT dried powder, the amounts of ferulic acid, calycosin, formononetin, Z-ligustilide, and total polysaccharide were determined: this was to ensure the chemical control of DBT (**Supplementary Table S1**). Calycosin and ferulic acid were the only varying parameters in DBT_Δcal_ and DBT_Δfa_, respectively (**Supplementary Table S1**). As compared with authentic DBT, a depletion of calycosin in DBT_Δcal_ was over 97%, and a depletion of ferulic acid in DBT_Δfa_ was over 98%.

**FIGURE 1:**
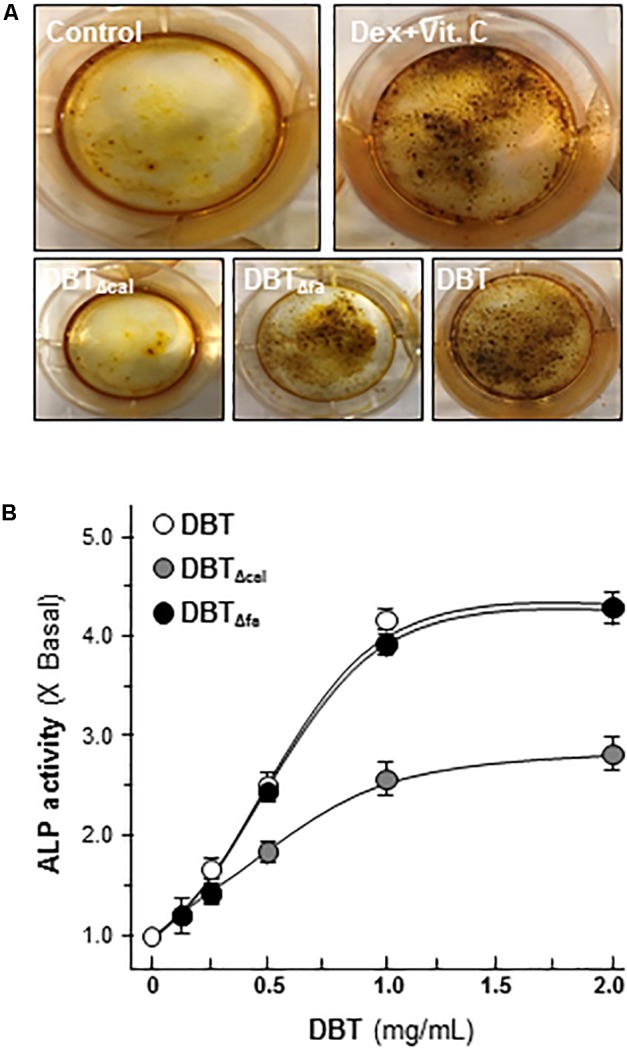
Danggui Buxue Tang (DBT) decoctions possess osteogenic functions. **(A)** Cultured osteoblasts were treated with different DBT (all at 1 mg/mL), or DBT_Δfa_, or DBT_Δcal_, for 21 days, and the stained nodules were found, as shown by Alizarin red staining. Representative images were shown. Dexamethasone plus vitamin C (Dex; 50 nM, Vit C; 250 μM) in the presence of β-glycerophosphate (5 mM) served as the positive control. **(B)** Different concentrations of DBT, or its chemical modified forms, were applied onto cultured osteoblasts for 7 days to analyze alkaline ophosphatase (ALP) enzymatic activity triggered by herbal decoctions. Data are expressed as the fold of increase compared with control (X basal), Means ± SEM, *n* = 4.

Osteoblast differentiation is an active and dynamic process, and many protein factors are being expressed at different differential stages. For example, ALP and Runx2 are the markers for earlier stage of differentiation, and the deposition of minerals is one of later indicative markers for osteoblastic differentiation (Lisberg et al., 2017). Here, cultured rat osteoblast was induced to differentiate under the treatments of dexamethasone plus vitamin C for 21 days. The authentic DBT, DBT_Δcal_, or DBT_Δfa,_ was applied onto cultured osteoblasts. Mineralization assay was carried out by Alizarin red staining method (**Figure 1A**). Besides calcium mineralization, the activity of alkaline phosphatase (ALP) was increased by application of DBT in a dose-dependent manner (**Figure 1B**). The abilities in inducing mineralization and ALP were decreased in DBT_Δcal_ but not for DBT_Δfa_ (**Figure 1B**), suggesting that calycosin was an indispensable chemical within DBT herbal decoction in stimulating bone formation. Nevertheless, the related signaling mechanisms remained unknown.

### Transcriptomics of DBT, DBT_Δcal,_ and DBT_Δfa_

**FIGURE 2:**
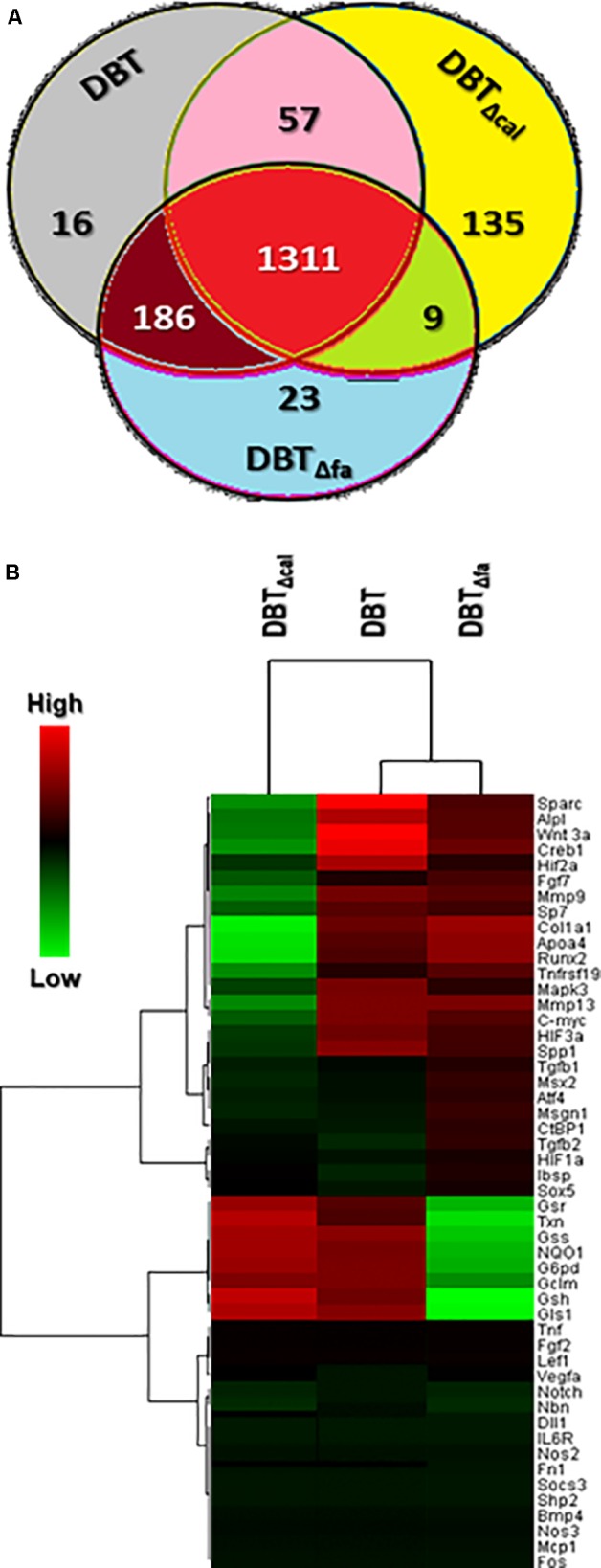
Venn diagrams and heatmap of RNA-seq results. **(A)** The over twofold up-regulated genes triggered by different herbal decoctions, i.e., DBT, DBT_Δfa,_ and DBT_Δca_ were shown here. The values indicated the number of genes stimulated by the decoctions. **(B)** Expression profiles of top 50 genes (see also **Supplementary Table S2**) induced by DBT were shown. Clustering software (version 3.17.1) was employed here to generate heat-map graph. Heat-map representation of mapped reads corresponds to protein-coding genes. The threshold was set at fivefold; the red color represented significantly increased; the green color indicated lower activation compared to untreated cells.

The transcript expressions of osteoblasts, treated with different herbal extracts, i.e., DBT, DBT_Δcal_ and DBT_Δfa_, were analyzed by RNA-seq. A VENN diagram was employed here to analyze the overlapping genes, activated by three DBT decoctions (**Figure 2A**). The twofold at least up-regulated genes, induced by three DBT decoctions, were selected for comparison. The results could be divided into seven sub-groups: 1,570 genes were up-regulated by DBT; 1,512 genes were up-regulated by DBT_Δcal_; and 1,529 genes were up-regulated by DBT_Δfa_ (**Figure 2A**). We found that 16 (∼1.0% of total) genes were solely activated by DBT; 135 (∼9.0%) genes were stimulated by DBT_Δcal_; 22 (∼1.5%) of genes were triggered by DBT_Δfa_ (**Figure 2A**). The up-regulated genes, triggered by the herbal extracts, were subjected to Heatmap software. The stronger activation was shown in red color, and the weaker stimulation was exhibited in green color; the threshold was set at a fivefold of increase. From the two-dimensional graphical data, the transcript profiles, triggered by authentic DBT and DBT_Δfa,_ shared a close similarity (**Figure 2B**). The transcript profile, triggered by DBT_Δcal_, showed a strong distinction as compared to that of DBT, or DBT_Δfa_, in particular for those genes having a robust regulation by application of DBT (**Figure 2B**). The top 50 genes, induced by DBT, or DBT_Δcal_, or DBT_Δfa_, were shown in **Supplementary Table S2**. The top on the list are those genes relating to osteoblastic differentiation, e.g., osteonectin (Sparc), Wnt3a, alkaline phosphatase (ALP), runt related transcription factor 2 (Runx2), osterix (Sp7), osteocalcin (Bglap).

**FIGURE 3:**
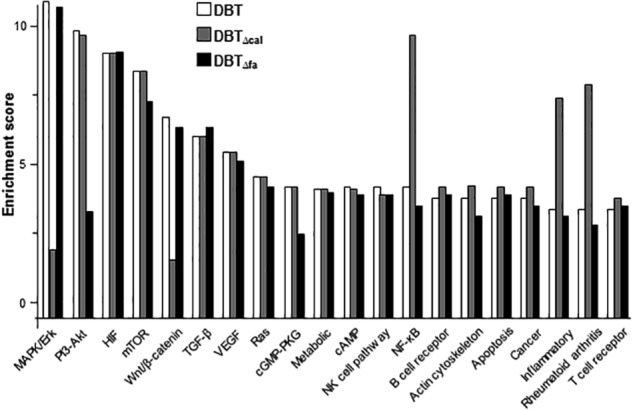
KEGG analysis of pathways induced by DBT. There were 20 pathways stimulated by herbal decoctions, including:MAPK signaling pathway; PI3-Akt signaling pathway; HIF signaling pathway; mTOR signaling pathway; Wnt signaling pathway; TGF-β signaling pathway; VEGF signaling pathway; Ras signaling pathway; cGMP-PKG signaling pathway; metabolic pathway; cAMP signaling pathway; natural killer cell mediated cytotoxicity; NF-κB signaling pathway; B cell receptor signaling pathway; regulation of actin cytoskeleton; apoptosis; pathway in cancer; inflammatory bowel diseases; rheumatoid arthritis; T cell receptor signaling pathway.

KEGG^3^ serves as a basic platform for the systematic analysis of gene functions in terms of the networks of gene products (Kanehisa et al., 2012). The up-regulated genes were subjected to KEGG analysis. The top 20 mechanisms, activated by DBT, were shown in **Figure 3**. In line to gene activation, the signaling pathways corresponding to bone formation, i.e., MAPK/Erk and Wnt/β-catenin, were markedly decreased in DBT_Δcal_-treated osteoblasts (**Figure 3**). Indeed, MAPK/Erk and Wnt/β-catenin signaling have been shown to be critical for the differentiation of osteoblasts. The pathways corresponding to inflammation however were markedly induced in DBT_Δcal_-treated osteoblasts. By comparing DBT and DBT_Δfa_, the pathways triggered in both cases were very similar, except a significant decrease in PI3-Akt signaling was revealed (**Figure 3**).

### Validation of RNA-Seq Results

**FIGURE 4:**
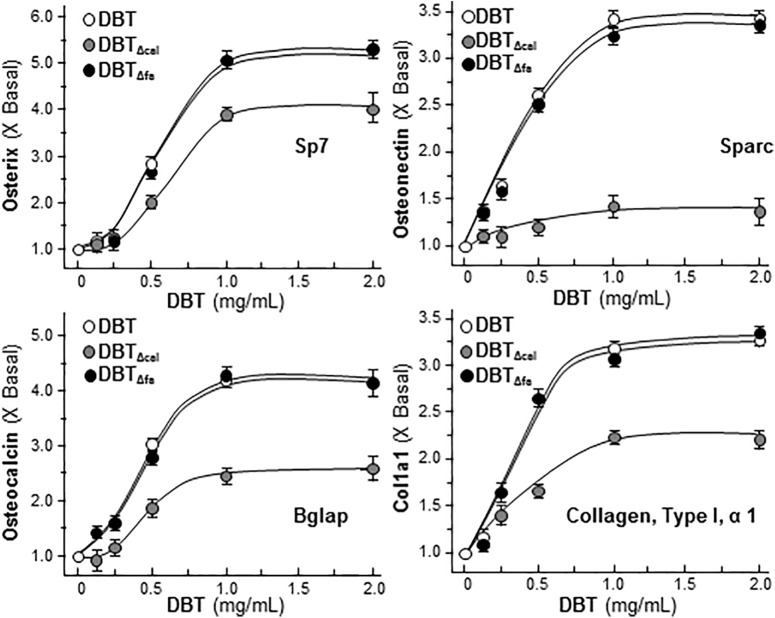
Reduced osteogenic activity under the treatment of DBT_Δcal._ Cultured cell was applied with a series of DBT decoctions at different concentrations for 7 days. The total RNA was extracted from the cultures and reversed transcribed into cDNA. The mRNA levels were analyzed by real-time PCR, i.e., osterix, ostenonectin, osteocalcin, and col1a1. GAPDH was used as an internal control. Values were expressed as the ratio to the basal reading where the control (untreated culture) equaled to 1 and in Mean ± SEM, where *n* = 4, each with triplicate.

The mRNA levels of late stage of osteoblastic differentiation markers, including Sp7 (osterix), Sparc (ostonectin), Bglap (osteocalcin) and Col1a1 (Collagen 1 type 1), induced by DBT, were revealed to be markedly reduced in treatment of DBT_Δcal_, as compared to that of authentic DBT (**Supplementary Table S2**). By real-time PCR analysis, these differentiation markers were up-regulated by DBT in a dose-dependent manner (**Figure 4**). The maximal activations of DBT-induced transcript expressions were from 3 to 5 folds starting from 1 mg/mL of DBT, and the dose response curves were rather similar (**Figure 4**). The transcript inductions of DBT_Δcal_ were much reduced (**Figure 4**). As compared to DBT, the induction triggered by DBT_Δcal_ was decreased by ∼20% in osterix, ∼90% in ostonection, ∼50% in osteocalcin, and ∼30% in Col1a1, respectively. The effects of DBT_Δfa_ were not altered as compared to that of DBT (**Figure 4**).

**FIGURE 5:**
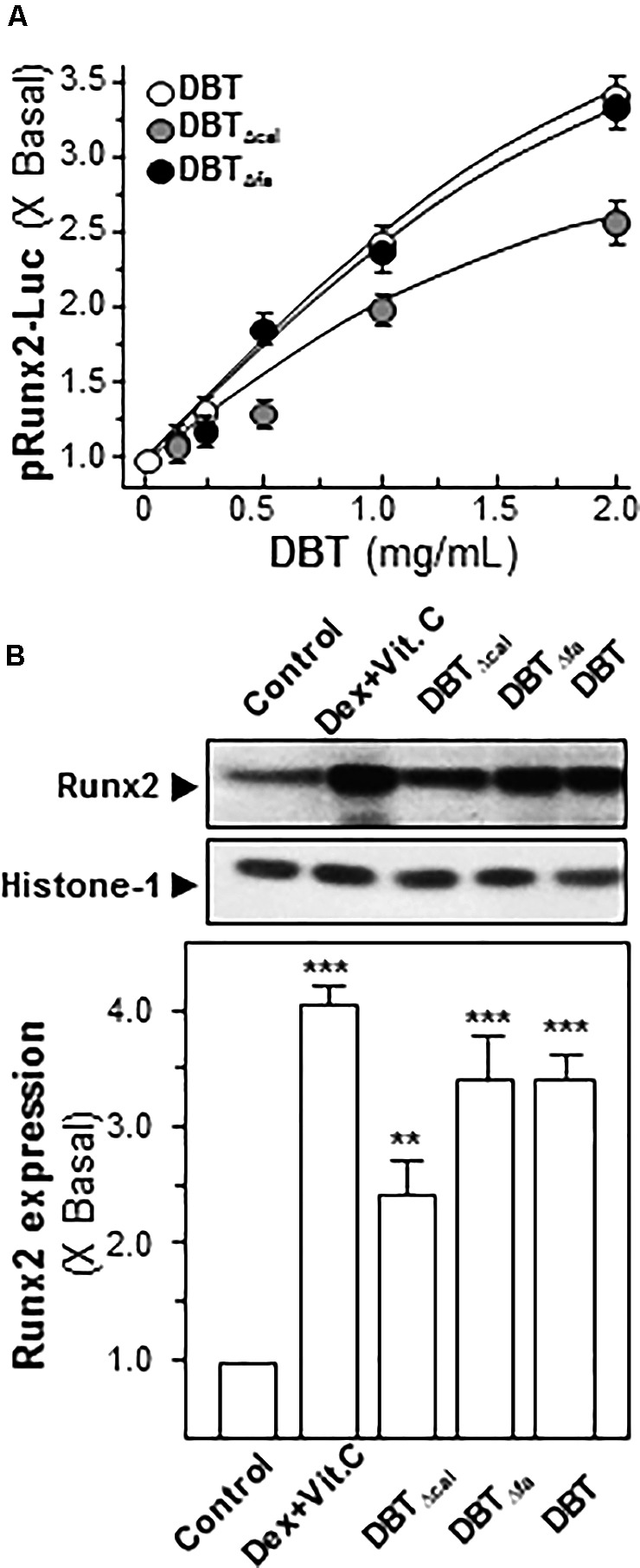
Activation of Runx2 expression requires calycosin in DBT.**(A)** In pRunx2-Luc transfected osteoblasts, the promoter activity was revealed by luciferase assay in cultured osteoblasts by treating a series of DBT decoctions at different concentrations for 7 days. Dexamethasone plus vitamin C (Dex; 50 nM, Vit C; 250 μM) was used as positive control, which induced ∼4-fold activation. **(B)** Cultured osteoblasts were treated with a series of DBT (1.0 mg/mL) for 7 days. Cell lysates were collected to determine the protein expression of Runx2 (∼57 kDa) using specific antibody. Histone-1 (∼30 kDa) served as a loading control. Quantification of protein amount from the blot was calculated by a densitometer. Values were expressed as the ratio to the basal reading, where the control (untreated culture) equaled to 1 and in Mean ± SEM, where *n* = 3. *^∗∗∗^p* < 0.001*, ^∗∗^p* < 0.01 as compared to the control.

Runx2 is a master transcriptional factor regulating differentiation of osteoblast. Here, transcriptional and translational levels of Runx2 were revealed in DBT-treated osteoblasts. The transcriptional activities of Runx2 were detected by luciferase assay. After application of DBT in cultured osteoblasts, the Runx2 promoter-driven luciferase (pRunx2-Luc) was increased in a dose-dependent manner, and DBT and DBT_Δfa_ showed similar profiles (**Figure 5A**). The activation of pRunx2-Luc activity, triggered by DBT_Δcal_, was markedly reduced at ∼30% less, as compared to that of DBT. In line to the transcriptional rate, the protein level of Runx2 (at ∼57 kDa) was regulated by DBT (**Figure 5B**). Again, the depletion of calycosin in DBT showed a weak induction, as compared to that of DBT. Thus, calycosin might be a critical chemical within DBT, rather than ferulic acid, in triggering osteogenic functions.

**FIGURE 6:**
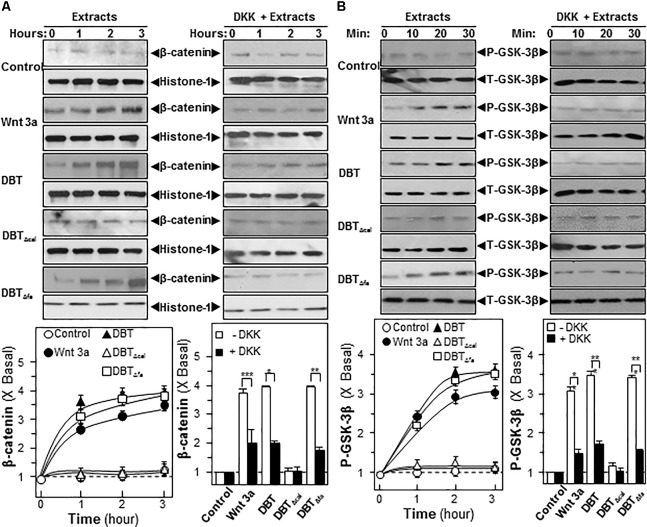
Danggui Buxue Tang and DBT_Δfa_ activate β-catenin translocation and GSK phosphorylations. Serum free osteoblasts were pre-treated with fresh medium, or 100 ng/mL of DKK, for 3 h before the application of 1.0 mg/mL of DBT for indicated time. **(A)** Beta-catenin (∼95 kDa) was detected by immunoblot analysis using specific antibodies, Histone-1 (∼30 kDa) served as an internal control. **(B)** Phosphorylation of GSK-3β (∼47 kDa) was detected by immunoblot analysis using specific antibodies, total GSK-3β (∼47 kDa) served as an internal control. Quantification of phospho-protein expression was calculated by a densitometer. Wnt3a (200 ng/mL) served as positive control. Values were expressed as the ratio to basal reading where the time zero (without treatment) equaled to 1, values were expressed as Mean ± SEM, where *n* = 3. *^∗∗∗^p* < 0.001*, ^∗∗^p <* 0.01, and *^∗^p* < 0.05 as compared to the control.

The signaling pathways of MAPK/Erk and Wnt/β-catenin were shown to be highly sensitive to calycosin in DBT, and thus which were validated here. β-Catenin is a master protein being regulated in Wnt pathway: one being activated, it can translocate from cytosol to nucleus in inducing the expression of canonical Wnt-targeted genes (Guo et al., 2012). Here, we examined the translocation of β-catenin in cultured osteoblast under the treatment of the herbal decoction. Like Wnt3a, application of DBT and DBT_Δfa_ in cultured osteoblasts induced the translocation of β-catenin in a time-dependent manner (**Figure 6A**). However, DBT_Δcal_ was not capable in triggering the translocation (**Figure 6A**). The application of DKK, a Wnt 3a antagonist, suppressed β-catenin translocation, as induced by Wnt3a, or DBT, or DBT_Δfa_ (**Figure 6A**). Protein kinases transfer phosphate groups from ATP to serine, threonine, or tyrosine residues on protein peptide substrates, directly affecting the activity and function of the target. Besides β-catenin translocation, we also determined the phosphorylation of GSK-3β in cultured osteoblast cells. Wnt3a, DBT, and DBT_Δfa_ induced the phosphorylation by ∼3-fold in a time-dependent manner, except DBT_Δcal_ (**Figure 6B**). As expected, the phosphorylation was markedly blocked by application of DKK (**Figure 6B**).

**FIGURE 7:**
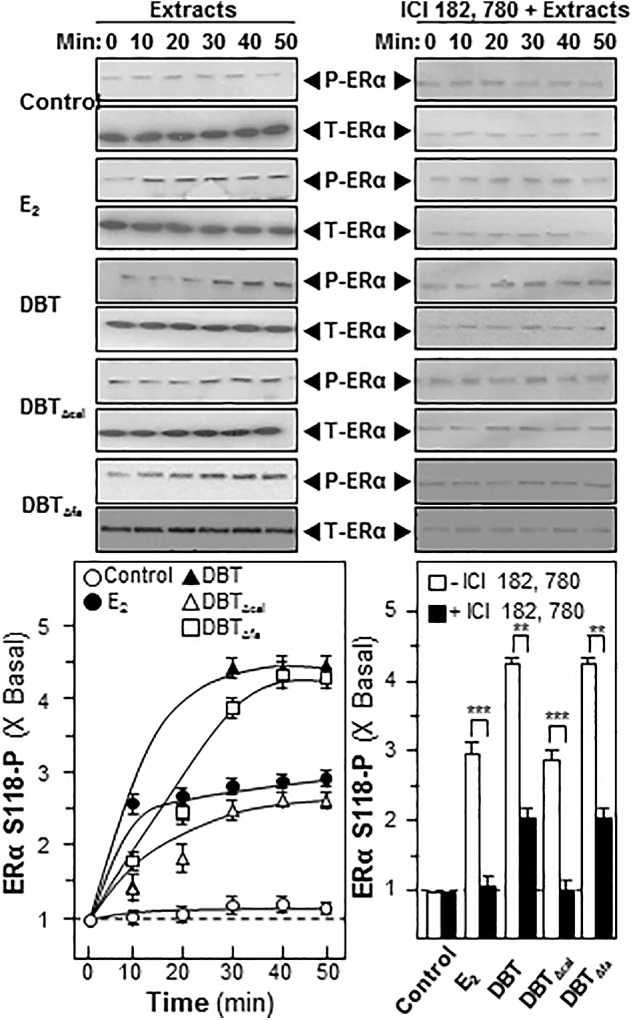
Danggui Buxue Tang induces estrogen receptor phosphorylation in cultured osteoblasts. Serum free cultured osteoblasts were pre-treated with fresh medium, or 100 nM of ICI 182,780, for 3 h prior to treatment of 1.0 mg/mL of DBT for indicated time. Phosphorylation of ER (∼60 kDa) was detected by immunoblot analysis using specific antibodies, total ER (∼60 kDa) served as internal control. Quantification of phospho-protein expression was calculated by a densitometer. E2 (100 nM) served as positive control. Values were expressed as the ratio to the basal reading where the time zero (without treatment) equaled to 1, values were expressed as Mean ± SEM, where *n* = 3. *^∗∗∗^p* < 0.001*, ^∗∗^p* < 0.01, and *^∗^p* < 0.05 as compared to the control.

Estrogen is a major inducer for bone differentiation acting via MAPK/Erk signaling (Choi et al., 2011). Here, we utilized western blotting to determine the ERα phosphorylation under the treatment of DBT. Authentic DBT and DBT_Δfa_ possessed strong activations on ERα phosphorylation; however, the induction was robustly reduced in the challenging of DBT_Δcal_ (**Figure 7**). The pre-treatment of ICI 182,780, as ER blocker, could significantly abolish the activation in all cases (**Figure 7**).

### Metabolomics Analysis

**FIGURE 8:**
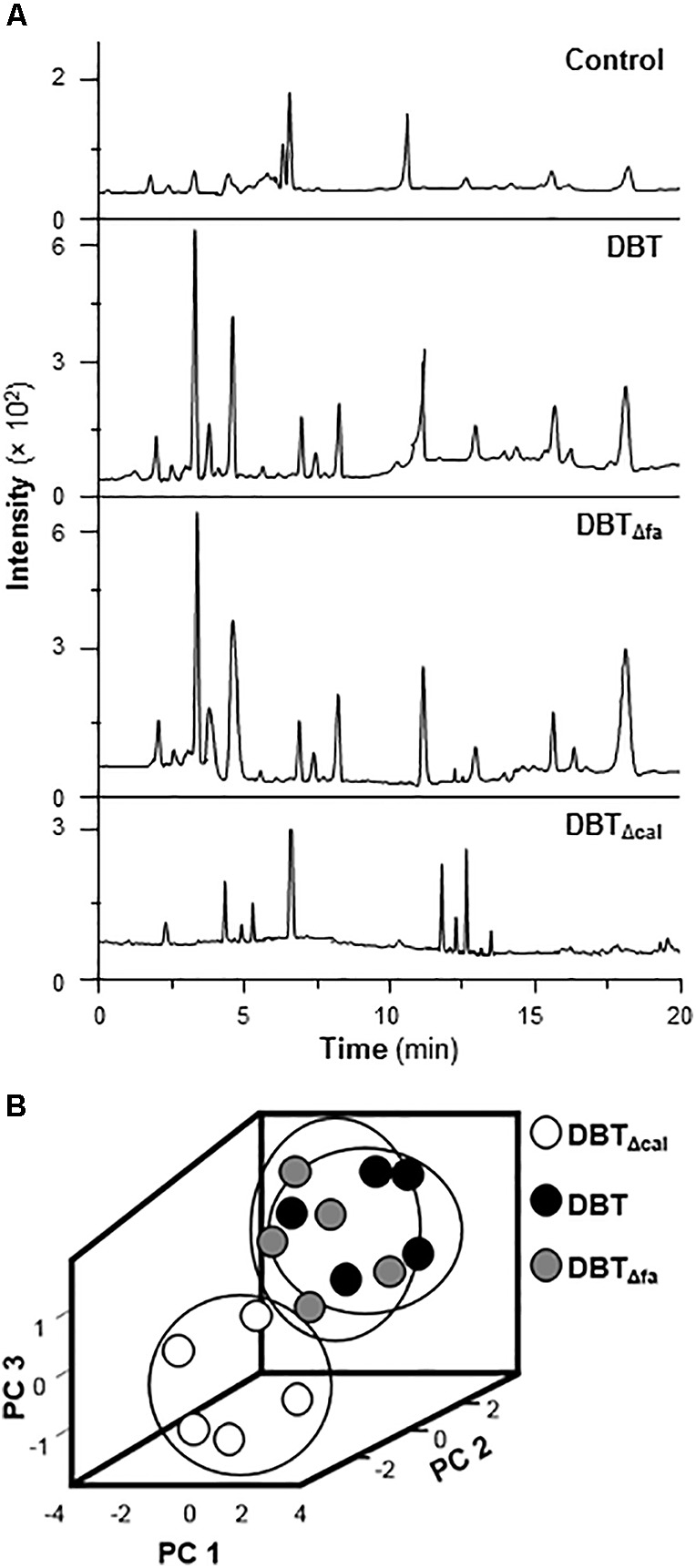
Metabolites and PCA analysis. **(A)** Typical LC–MS/MS spectra of metabolites. Cell lysate was collected after drug treatment for 7 days and then performed analysis. Five μL of samples were injected for LC–MS/MS. Representative chromatograms are shown, *n* = 5. **(B)** The score plot of biological properties from different DBT metabolites. The input materials were the peak area of each metabolites and retention time.

Metabolomics is another parameter to explore the working mechanism or related metabolites under the treatment of TCM in cultured cells. Cultured rat osteoblasts were treated with 1 mg/mL of DBT, DBT_Δcal,_ or DBT_Δfa_, for 7 days, as that in transcriptomics study. After extensive washing, the cell lysate was collected and subjected to LC–MS/MS analysis. Each peak represented at least one metabolite, and therefore the metabolites, under the applications of DBT and DBT_Δfa_, shared a great similarity (**Figure 8A**). Distinct profile was revealed for the treatment of DBT_Δcal_. PCA provides a roadmap to show how a complex data set can be transformed to a lower dimension to reveal the differences of various samples (Chan et al., 2014). The loading plots of PCA reveal whether the biological data contribute significantly to the inter-group differences in which they are at the farthest from main cluster of analyzed materials. PCA was employed to explore the differences of metabolites, generated from osteoblasts treated with different DBT decoctions (**Figure 8B**). DBT_Δcal_ showed a very distinct metabolic profile, as compared to that of DBT, or DBT_Δfa_. The chemical profiles of authentic DBT and DBT_Δcal_ showed the great similarities, except from amount of calycosin. Thus, calycosin should act as an essential ingredient within DBT decoction in regulating the metabolites of osteoblasts.

## Discussion

A TCM herbal formula normally composes of at least two or more herbs, and which could be modified/revised according to the character of patient. Because of chemical complexity, the action mechanism of any TCM formula is not known (Qu et al., 2003; Ma and Jia, 2011; Zhang et al., 2012; Lin et al., 2017). The unknown action mechanism of TCM formula therefore is an obstacle for general acceptance of TCM by western countries (Qu et al., 2003; Ma and Jia, 2011). Unfortunately, TCM is not able to show clear information about the exact efficacy of individual chemical(s); because a full chemical profile of any TCM extract is still not known. Here, a selective chemical knock-out method was designed to solve this problem. This method was utilized to deplete those interesting bioactive chemicals from a herbal mixture. Therefore, the correlations between active chemicals within a decoction and its detailed underlying mechanisms could be elucidated. In addition, this methodology created a platform to study the compatibilities of one chemical with others within a herbal mixture. Using the depletion of calycosin and ferulic acid here in DBT, we established an example to show an indispensable role of calycosin in function of osteogenesis, triggered by DBT. In absence of calycosin, DBT showed weak activity in inducing osteoblastic differentiation. Calycosin alone exhibited sluggish functions in motivating mineralization and Runx2 expression of cultured osteoblasts, as compared to DBT decoction (**Supplementary Figure S2**). This result is fully supportive to a hypothesis of each component has their unique role within the herbal mixture.

Steroid hormone, especially estrogen, is well-known for ERT in the treatment of osteoporosis (Choi et al., 2011). The side effects of ERT have been reported to increase the risk of developing breast cancer (Gong et al., 2016). Phytoestrogens are now popular for patients with better safety. Flavonoids share the major functional group of phytoestrogens, and they are believed to be one cluster of phytoestrogens. Indeed, they contained over 6,000 compounds and highly abundant in plants (Ferrer et al., 2008). Consuming of phytoestrogens could reduce the risk of cancer, and therefore they are believed to be one of indispensable nutritional compounds in daily diet (Tang et al., 2010). Apart from the osteogenic functions, calycosin also exhibited cardiovascular protective functions, anti-inflammation and induction of erythropoietin production (Zheng et al., 2011).

Transcriptomics analysis had been introduced to TCM study, and which provided a technological method to explore gene transcriptional profile triggered by a chemical standardized herbal decoction (Ren et al., 2013). Together with chemical knock-out method as described here in DBT, the bioinformatics results not only demonstrate the action mechanism triggered by DBT in stimulating osteoblastic differentiation; but which also provide a series of biomarkers that are specifically triggered by calycosin, or ferulic acid, within DBT decoction. Being illustrated here in cultured osteoblasts, several key osteoblastic biomarkers were specifically sensitive to calycosin in DBT, e.g., ALP, osterix and osteonectin. Indeed, these biomarkers are well-known playing key steps in differentiation of osteoblasts (Spencer et al., 2006; Zhang et al., 2017). Thus, this chemical knock-out plus transcriptomics methodology could be used for other herbal mixtures, as to reveal its action mechanism.

By signaling cascades analysis, the authentic DBT and DBT_Δfa_ were capable of inducing osteogenesis by multiple mechanisms, i.e., MAPK/Erk and Wnt/β-catenin pathways; DBT_Δcal_ was not capable of stimulating MAPK/Erk to induce bone formation. Wnt/β-catenin pathway is an important mechanism in monitoring osteoblast formation and differentiation through multiple mechanisms. Osteoblasts are originated from bone marrow mesenchymal stem cells. During the early stage of differentiation, Wnt/β-catenin signaling plays an important role in triggering the differentiation of bone marrow mesenchymal stem cells to osteoblasts. From the results, it showed that authentic DBT and DBT_Δfa_ triggered Wnt/β-catenin signaling cascades in regulating osteogenesis, including: (i) phosphorylation of GSK-3β; (ii) β-catenin nuclear translocation activity; and (iii) the blockage by application of DKK-1. Therefore, it indicated that calycosin played an essential role in monitoring osteoblastic differentiations, instead of ferulic acid from the decoction. By metabolomics analysis, we found the metabolites of treated cells of authentic DBT differed from DBT_Δcal_. Quantitative differences in the levels of metabolites, triggered by different DBT, might contribute to differences in pharmacological functions.

In addition to the study in osteoblasts, we have demonstrated the functional role of calycosin in calycosin-depleted DBT. DBT_Δcal_ lost its functions in regulating estrogenic and erythropoietic properties in cultures (Gong et al., 2015). In all cases, calycosin alone did not show any functions of that of DBT, at least in a dose that was corresponding to DBT at 1 mg/mL. Together with the current study, we aim to hypothesize that calycosin is acting as an organizer in orchestrating multi-components of DBT as to achieve maximal function for bone formation.

## Author Contributions

AG and KT conceived and designed the experiments, as well as wrote the main text. RD, HW, and TD contributed reagents/materials/analysis tool and identified the quality of herbs.

## Conflict of Interest Statement

The authors declare that the research was conducted in the absence of any commercial or financial relationships that could be construed as a potential conflict of interest.

## Footnotes

1 http://david.abcc.ncifcrf.gov/

2 http://kegg.org/

3 http://www.genome.jp/kegg/kegg1a.html

## Supplementary Material

FIGURE S1 Chemical and biological analysis of different DBT decoctions. **(A)** Typical HPLC fingerprint of chemical-modified DBT decoctions at 254 nm. One hundred mg/mL of parental DBT, DBT_Δcal_, and DBT_Δfa_ was subjected to HPLC analysis, and the chemical fingerprints were revealed at the wavelength 254 nm. **(B)** The chemical profiles were compared between DBT and DBTHPLC by LC–MS. **(C)** Different concentrations chemically modified DBT decoctions (0.125–2 mg/mL) were applied onto cultured cells for analyzing the enzymatic activity of ALP. Calycosin was re-added onto DBT_Δcal_ (as indicated) as to generate different percentage of calycosin depletion. Dexamethasone plus vitamin C (Dex; 50 nM, Vit C; 250 μM) was used as positive control. Values were expressed as the ratio to the basal reading where the control (untreated culture) equaled to 1 and in Mean ± SEM, where *n* = 4, each with triplicate.

FIGURE S2 Osteogenic functions triggered by calycosin and ferulic acid. **(A)** Cultured osteoblasts were treated with 693 ng of calycosin and 809 ng of ferulic acid (presented in 1 mg of DBT) for 3 weeks, and the stained nodules were found, as shown by Alizarin red staining. Representative images were shown. Bar = 5 mm. **(B)** Different concentrations of calycosin and ferulic acid were applied onto cultured osteoblasts for 7 days to analyze transcriptional activity of Runx2. Data are expressed as the fold of increase compared with control (X basal), Mean ± SEM, *n* = 4.

TABLE S1 Quantification analysis of each chemical within DBT decoctions.

TABLE S2 The top 50 genes triggered by DBT decoction.
