# Supplementary figures and images for: Calycosin Orchestrates Osteogenesis of Danggui Buxue Tang in Cultured Osteoblasts: Evaluating the Mechanism of Action by Omics and Chemical Knock-out Methodologies
Source: Front Pharmacol. 2018 Feb 1;9:36. doi: 10.3389/fphar.2018.00036 (PMC5799702; doi:10.3389/fphar.2018.00036)

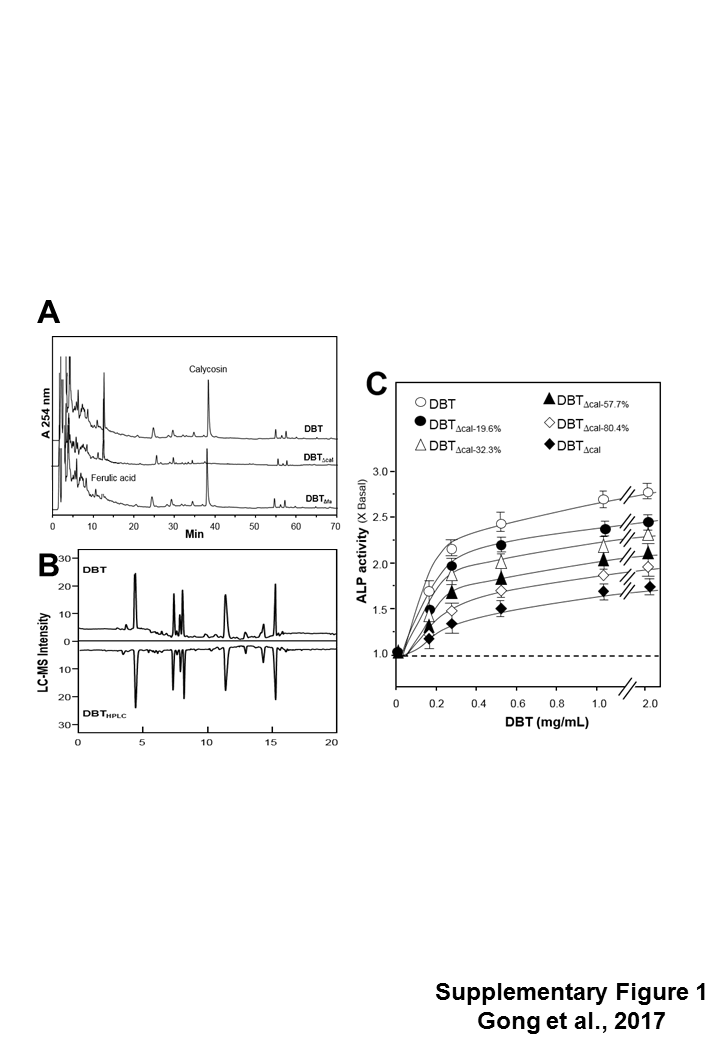

Supplement: FIGURE S1 — Chemical and biological analysis of different DBT decoctions. (A) Typical HPLC fingerprint of chemical-modified DBT decoctions at 254 nm. One hundred mg/mL of parental DBT, DBTΔcal, and DBTΔfa was subjected to HPLC analysis, and the chemical fingerprints were revealed at the wavelength 254 nm. (B) The chemical profiles were compared between DBT and DBTHPLC by LC–MS. (C) Different concentrations chemically modified DBT decoctions (0.125–2 mg/mL) were applied onto cultured cells for analyzing the enzymatic activity of ALP. Calycosin was re-added onto DBTΔcal (as indicated) as to generate different percentage of calycosin depletion. Dexamethasone plus vitamin C (Dex; 50 nM, Vit C; 250 μM) was used as positive control. Values were expressed as the ratio to the basal reading where the control (untreated culture) equaled to 1 and in Mean ± SEM, where n = 4, each with triplicate. [file Image_1.TIF]

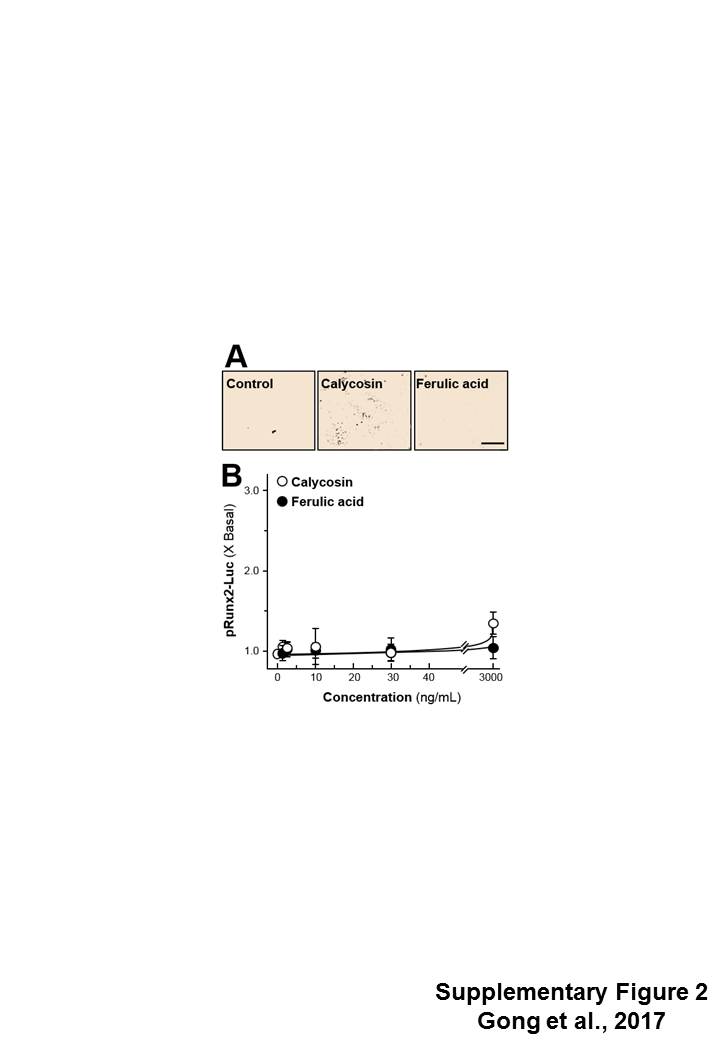

Supplement: FIGURE S2 — Osteogenic functions triggered by calycosin and ferulic acid. (A) Cultured osteoblasts were treated with 693 ng of calycosin and 809 ng of ferulic acid (presented in 1 mg of DBT) for 3 weeks, and the stained nodules were found, as shown by Alizarin red staining. Representative images were shown. Bar = 5 mm. (B) Different concentrations of calycosin and ferulic acid were applied onto cultured osteoblasts for 7 days to analyze transcriptional activity of Runx2. Data are expressed as the fold of increase compared with control (X basal), Mean ± SEM, n = 4. [file Image_2.TIF]
