# Supplementary material for: Calycosin Orchestrates Osteogenesis of Danggui Buxue Tang in Cultured Osteoblasts: Evaluating the Mechanism of Action by Omics and Chemical Knock-out Methodologies
Source: Front Pharmacol. 2018 Feb 1;9:36. doi: 10.3389/fphar.2018.00036 (PMC5799702; doi:10.3389/fphar.2018.00036)
Supplement: TABLE S1 — Quantification analysis of each chemical within DBT decoctions. [file Table_1.PDF]

Marker chemical[a]	Samples		
	DBT	$DBT_{\Delta cal}$[b]	$DBT_{\Delta fa}$
Ferulic acid	809.44[c]	806.44	12.14 ***
Calycosin	693.19	19.40 ***	692.05
Formononetin	164.58	166.02	164.12
Z-Ligustilide	212.01	213.83	212.73
Polysaccharides	13.49	13.77	12.98

Supplementary Table 1. Quantification analysis of each chemical within DBT decoctions

[a] Five chemicals were selected as marker chemicals, and which were determined by HPLC method and anthrone-sulfuric acid method for polysaccharide. These chemicals set parameters for minimal requirement for quality control.

[b] $DBT_{\Delta cal}$ or $DBT_{\Delta fa}$ was generated as stated in the method.

[c] Values were expressed in μg/g dried extract of DBT, except the amount of polysaccharide was in % of herbal extract, in Mean ± SEM, where $n = 5$. ***$p < 0.001$ as compared with parental DBT.
