# Supplementary material for: Calycosin Orchestrates Osteogenesis of Danggui Buxue Tang in Cultured Osteoblasts: Evaluating the Mechanism of Action by Omics and Chemical Knock-out Methodologies
Source: Front Pharmacol. 2018 Feb 1;9:36. doi: 10.3389/fphar.2018.00036 (PMC5799702; doi:10.3389/fphar.2018.00036)
Supplement: TABLE S2 — The top 50 genes triggered by DBT decoction. [file Table_2.PDF]

NO.	Genes	DBT	$DBT_{\Delta fa}$	$DBT_{\Delta cal}$	Gene ID	NO.	Gene	DBT	$DBT_{\Delta fa}$	$DBT_{\Delta cal}$	Gene ID
**1**	Sparc	9.3	7.9	2.4	24791	**26**	Bmp4	5.1	4.9	5.2	25296
**2**	Wnt3a	8.9	8.2	2.6	303181	**27**	Vegfa	5	5.2	4.9	83785
**3**	Alp	8.2	7.9	2.5	25586	**28**	Sox5	5	5	5	140587
**4**	Mmp9	7.1	6.7	3.9	81687	**29**	Socs3	5	4.9	5	89829
**5**	Runx2	6.7	7.5	2.6	367218	**30**	Shp2	5	5.1	5.2	25622
**6**	Col1a1	6.6	7.4	2.2	29393	**31**	Msx2	5	5.1	4.7	25483
**7**	Osterix	6.5	6.3	3.6	300260	**32**	Msgn1	5	5.1	4.6	689864
**8**	HIF3a	6.4	5.4	4.3	64345	**33**	Fos	5	5.2	4.9	314322
**9**	Apoa4	6.4	7.5	2.4	25080	**34**	CtBP1	5	5.2	5	29382
**10**	Spp1	6.3	5.3	4.4	25353	**35**	Atf4	5	5.1	4.6	79255
**11**	GCLM	6	3.5	6.9	29739	**36**	Nbn	4.9	5.2	5.1	85482
**12**	Mmp13	5.9	5.9	3.3	171052	**37**	Mcp1	4.9	5.2	5.1	24770
**13**	Mapk3	5.9	5.2	4	50689	**38**	IL6R	4.9	4.9	4.9	24499
**14**	G6pd	5.9	2.9	6.9	24377	**39**	HIF1a	4.9	5.2	5	29560
**15**	C-myc	5.9	5.4	4.3	24577	**40**	Fn1	4.9	5	4.9	25661
**16**	NQO1	5.6	2.1	7.2	24314	**41**	Fgf7	4.9	6.3	3.5	29348
**17**	Gls1	5.3	2.2	7.3	172682	**42**	Dll1	4.9	4.9	4.9	84010
**18**	Tnfrsf19	5.2	6	3.4	290300	**43**	Tgbf2	4.8	5.3	4.8	30701
**19**	Tgfb1	5.2	5.2	4.9	59086	**44**	Lef1	4.8	4.6	5.2	161452
**20**	Nos3	5.2	5.1	5	24600	**45**	Ibsp	4.8	5.1	5	24477
**21**	Hif2a	5.2	6	4	29452	**46**	Fgf2	4.8	4.8	5.2	54250
**22**	Gss	5.2	2.2	6.9	25458	**47**	Notch	4.4	5.1	4.9	25496
**23**	GSH	5.2	2.3	7	2937	**48**	Txn	3.6	4.2	3.9	7295
**24**	Tnf	5.1	4.9	5.2	24835	**49**	Gsr	3.2	4	4	116686
**25**	Nos2	5.1	4.9	4.8	24599	**50**	Creb	2.9	7.1	4.1	81646

Supplementary Table 2. The top 50 genes triggered by DBT decoction.

The quantification results of top 50 genes triggered by DBT decoctions. Values were expressed as the ratio to the basal reading, where the control (untreated culture) equaled to 1.
